# Graveyards on the Move: The Spatio-Temporal Distribution of Dead Ophiocordyceps-Infected Ants

**DOI:** 10.1371/journal.pone.0004835

**Published:** 2009-03-12

**Authors:** Maj-Britt Pontoppidan, Winanda Himaman, Nigel L. Hywel-Jones, Jacobus J. Boomsma, David P. Hughes

**Affiliations:** 1 Section for Ecology and Evolution, Department of Biology, University of Copenhagen, Copenhagen, Denmark; 2 Forest Entomology and Microbiology Group, National Park, Wildlife and Plant Conservation Department, Bangkok, Thailand; 3 Mycology Laboratory, National Center for Genetic Engineering and Biotechnology, Science Park, Pathum Thani, Thailand; 4 Centre for Social Evolution, Department of Biology, University of Copenhagen, Copenhagen, Denmark; 5 Museum of Comparative Zoology Labs, Department of Organismic and Evolutionary Biology, Harvard University, Cambridge, Massachusetts, United States of America; 6 School of Biosciences, University of Exeter, Exeter, United Kingdom; University of Arizona, United States of America

## Abstract

Parasites are likely to play an important role in structuring host populations. Many adaptively manipulate host behaviour, so that the extended phenotypes of these parasites and their distributions in space and time are potentially important ecological variables. The fungus *Ophiocordyceps unilateralis*, which is pan-tropical in distribution, causes infected worker ants to leave their nest and die under leaves in the understory of tropical rainforests. Working in a forest dynamic plot in Southern Thailand we mapped the occurrence of these dead ants by examining every leaf in 1,360 m^2^ of primary rainforest. We established that high density aggregations exist (up to 26 dead ants/m^2^), which we coined graveyards. We further established that graveyards are patchily distributed in a landscape with no or very few *O. unilateralis*-killed ants. At some, but not all, spatial scales of analysis the density of dead ants correlated with temperature, humidity and vegetation cover. Remarkably, having found 2243 dead ants inside graveyards we only found 2 live ants of the principal host, ant *Camponotus leonardi*, suggesting that foraging host ants actively avoid graveyards. We discovered that the principal host ant builds nests in high canopy and its trails only occasionally descend to the forest floor where infection occurs. We advance the hypothesis that rare descents may be a function of limited canopy access to tree crowns and that resource profitability of such trees is potentially traded off against the risk of losing workers due to infection when forest floor trails are the only access routes. Our work underscores the need for an integrative approach that recognises multiple facets of parasitism, such as their extended phenotypes.

## Introduction

Parasites negatively affect their hosts in multiple ways. Because disease prevention and containment is crucial in many arenas such as city living, agriculture and the conservation of endangered species, we know a great deal about parasite spatio-temporal dynamics [1]. In such an epidemiological approach, where the goal is to understand transmission dynamics the key information that is recorded is the location of the infected hosts in a population of healthy individuals and the history of transmission prior to the current sampling date [2]. This is a host centred approach to understanding parasite spatio-temporal dynamics. However, in certain host-parasite systems uninfected individuals may be completely absent in some populations because the parasites have manipulated the behaviour of the infected hosts so that they aggregate [3]. Often this is a parasite adaptation to increase fitness and so is viewed as an extended phenotype of the parasite [4]. This is an explicitly parasite centred approach and it has not been generally considered when examining parasite spatio-temporal dynamics.

Many examples of parasites altering host behaviour exist [3] and a few choice ones illustrate the often dramatic effects observed: nematodes and nematomorphs cause various insect hosts (e.g. crickets, ants) to drown themselves so the adult parasite can reproduce in water [5], [6]; parasitoids cause bees to bury themselves alive [7] or spiders to build aerial cocoons so as protect the developing parasitoid pupa [8] and many arthropods, fish and mammals have altered behaviour that makes it much easier for predators to catch them which enables the parasite to be passed on trophically [3], [9]. The field of parasite manipulation is currently going through interesting and significant changes as researchers move beyond merely cataloguing abnormal behaviour in infected individuals [10]. For example, determining the proximate mechanisms by which such changes are induced [11]; the effects of aggregated hosts on biodiversity [12] or intraspecific competition [13]. Here we want to add the spatio-temporal distribution of behaviourally manipulated hosts as an additional direction. We will use Carpenter ants (genus *Camponotus*) infected by the behaviourally manipulating fungal parasite, *Ophiocordyceps unilateralis* as a model.

**Figure 1:**
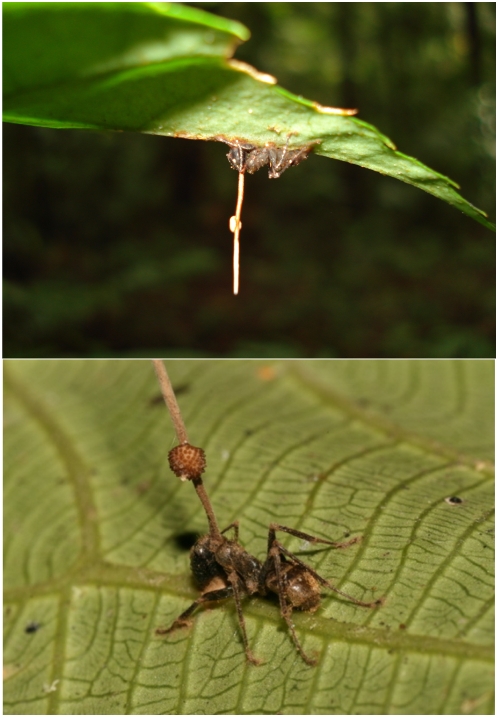
Dead ants infected with *Ophiocordyceps unilateralis*. Ants biting the underside of leaves as a result of infection by *O. unilateralis*. The top panel shows the whole leaf with the dense surrounding vegetation in the background and the lower panel shows a close up view of dead ant attached to a leaf vein. The stroma of the fungus emerges from the back of the ant's head and the perithecia, from which spores are produced, grows from one side of this stroma, hence the species epithet. The photograph has been rotated 180 degrees to aid visualization.

Ants infected by the fungus *O. unilateralis* die in a dramatic way [14], [15], [16], [17]. Ants, when foraging, are infected by fungal spores that adhere to, and then penetrate their cuticle. The period of infection may be as short as 3–6 days [15] and once the infected ant is dead the fungus produces a large stalk, growing from the back of the ant's head (fig. 1). From this structure spores are released onto the forest floor where they infect new hosts. Spores are too large to be wind dispersed and instead fall directly to the ground where they produce secondary spores that infect ants as they walk over them [18]. Infected ants may die in various locations such as under leaves, on tree bark and attached to stems [15]. Dying is preceded by biting behaviour where ants clamp onto plant surfaces. It is this biting behaviour that is the extended phenotype of the fungus because biting maintains the ant in situ after the ant dies giving the fungus time to produce an adhesive that more permanently binds the ants to the plant (fig. 1). A remarkable feature of tropical forests is that very high densities of dead ants can occur leading to a phenomenon we term graveyards. These have been anecdotal recordings in South America [16], [17], Africa [14] and Australasia (Hywel-Jones, personal observations).

The purpose of this study was to systematically survey the spatio-temporal distribution of dead *O. unilateralis*-infected ants in a primary tropical forest in Southern Thailand. On the basis of limited previous observations [14], [16], [17] we expected to find patchy aggregations of ants infected with *Ophiocordyceps*. We wanted to test the hypothesis that graveyards occur and determine if the density of ants within them is correlated to biotic and abiotic variables. We further wanted to determine if uninfected ants occur in graveyards. Because fungal diseases produce flushes of dead insects [19] we also wanted to test if graveyards are an ephemeral phenomenon in tropical rainforests.

### Taxonomic note

In Thailand, regular surveys at 42 sites over the last 15 years have confirmed that *O. unilateralis* only infects ants, that it is the most common entomopathogenic fungus in Thai forests, and that ants die under leaves [20]. Other insects, as well as ants, are infected by different species of *Ophiocordyceps* [18]. The species *O. unilateralis* is pan-tropical and can be found causing similar behavioural changes in Africa and South America [14], [15], [16], [17], [21]. All species in the genus *Ophiocordyceps* were formerly known as *Cordyceps* but have recently been shifted to the family *Ophiocordycipitaceae* and the genus *Ophiocordyceps* was re-erected [18].

## Results

### Host range & ant foraging

All dead ants found biting the underside of leaves were infected with the species *O. unilateralis* (or its asexual state, *Hirsutella formicarum*: field determination of both states is possible because of the very characteristic appearance of this fungus). We counted 2243 dead ants inside the plots. Visual identification of ants in situ under leaves revealed that most ants were workers of the *Camponotus leonardi*. When 100 of these ants were examined in the laboratory with a stereomicroscope 3 proved to belong to the closely related genus *Polyrhachis* (both genera are in the Formicinae sub-family). The ant, *C. leonardi* is the principal host of the fungus *O. unilateralis* in Thailand and our field collections are confirmed by the high occurrence of this host species in the 3000+ historical records at the Herbaria of BIOTEC, Bangkok, which stretch back 15 years (Gerritsma, Hywel-Jones & Hughes, unpublished data). In spite of intensive searching during 5 weeks (estimated to be more than 500 person hours) no *C. leonardi* colonies or trails were found in the survey area and only two living *C. leonardi* ants were encountered in the plots during counting. Other ants from a wide range of genera and sub-families were abundant both during daylight and night-time surveys. In addition to visual searching for *C. leonardi* ants we also used food baits, adhesive traps and pitfall traps but no *C. leonardi* ants were captured.

However, 21 days after fieldwork began an active trail of *C. leonardi* was found in the vicinity of plot 3 and 4. The trail descended from a large tree, and went up into another large tree after ca. 5 m. It later descended again ca. 20 m further on before re-ascending into the canopy. Later visits to the field site (September 2007) found a single *C. leonardi* nests 20–25 m above the forest floor near plot 5. Aerial highways of ants extended 50–100 m across the canopy. We thus conclude that the species *C. leonardi*, and not other species of ants, is the principal host of *O. unilateralis* at our site and that ant trails are rarely found at ground level but more abundantly in the canopy where the colonies are located. Other surveys of other insect parasitic fungi at our site (September, 2006, January 2007, March 2007, September 2008) revealed a low overall diversity of other *Ophiocordyceps* species (Hywel-Jones et al, unpublished). In those surveys all potential habitats are surveyed (soil, decaying wood, tree trunks, stems and leaves) so we can be certain that our focus on sampling dead ants under leaves has not overlooked areas where ants or other insects killed by *O. unilateralis* could occur.

### Spatial patterns

**Figure 2:**
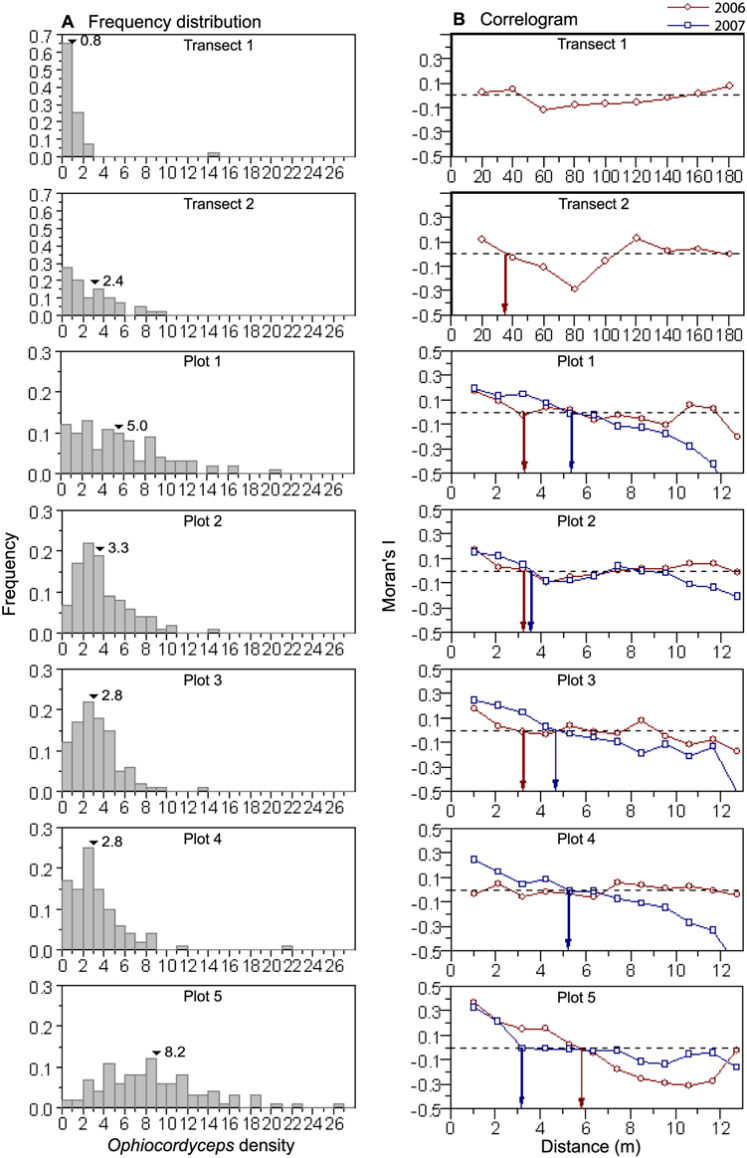
Density frequency distributions and Moran's I correlograms. a. Density frequency distributions for the five plots and the two transect in 2006. Mean *Ophiocordyceps* density is indicated with an arrow. b. Moran's I values plotted against distance classes for 2006 (blue) and 2007 (red). Positive I values indicate a positive autocorrelation while I values around zero indicate absence of autocorrelation. Arrows show the distances where the curves intercept the zero-line, an intercept that estimates the average patch diameter.

Counts of dead *O. unilateralis* infected ants in transect plots revealed that plots chosen without prior knowledge of the density of dead ants, contained no or only a few *O. unilateralis* infected ants in most cells, creating strongly right-skewed frequency distributions (Fig. 2a). In transect 1 the mean density was 0.8 ants/m^2^; 65% of the 40 cells were empty and only one cell contained more than 3 ants. Transect 2 had a somewhat higher mean density of 2.4 ants/m^2^, but still 73% of the cells (i.e. 29 cells) contained less than 4 ants. However, within this almost *O. unilateralis* free environment we also found patches of higher density. In transect 1, a single cell of 1 m^2^ contained 14 dead ants and in transect 2, we found 10% of the cells containing more than 6 dead ants.

The smaller scale patterns obtained from the five 100 m^2^ sampling plots within the graveyards gave a different impression than the large scale pattern encountered from the two transects. Recall that the five sampling plots were chosen after identifying the area as a high density location. Confirming that we chose graveyard locations we found much higher mean densities here, namely 8.2 ants/m^2^ in plot 5 and 5.0 ants/m^2^ in plot 1, 3.3 ants/m^2^ in plot 2, and 2.8 ants/m^2^ in both plot 3 and 4. In contrast to the transect plots, the five sampling plots also had less right-skewed density frequency distributions (Fig. 2a). Cell counts of *O. unilateralis* infected ants ranged from 0 to 26 ants but only between 2 and 17% of the cells contained no individuals.

Parametric tests on log-transformed densities showed that transect plots were significantly different from sampling plots (t-test, df = 578, t = 6.13, p<0.01), confirming that the five sampling plots indeed were situated in patches where densities of *O. unilateralis* were higher than those found in the transect areas chosen without prior knowledge of densities. ANOVA also showed significant differences between the five sampling plots (F_4,495_ = 33.23, p<0.01), and a subsequent Tukey test showed plot 5 to be different from all other plots and plot 1 to be different from plot 3, 4 and 5.

The correlogram found Transect 1 to have Moran's I values around zero for all distance classes, thus exhibiting no autocorrelation (Fig. 2b). Taking the low mean density into account, this suggests that transect 1 ran through a nearly *O. unilateralis* free environment with very few randomly scattered dead ants. In contrast, the correlogram for transect 2 showed a more patchy pattern with weak autocorrelations at the lowest distance class (I = 0.12, p = 0.04) and again at 120 m (I = 0.13, p = 0.18), suggesting that transect 2 ran through two aggregations of dead *O. unilateralis* infected ants approximately 100 m apart. The two peaks in Moran's I values reached zero again after 25–30 m indicating that patches had a considerably smaller diameter than the distance between them (Fig. 2b). Taken together, the correlograms showed that dead *O. unilateralis* infected ants did not appear randomly distributed, but were aggregated in large patches (graveyards) within an otherwise nearly *O. unilateralis* free environment.

The five sampling plots were located within such graveyards and the correlograms also showed that the graveyards themselves were spatially structured. In all plots, except plot 4, positive autocorrelations were found at the lowest distance classes (1 m) (Plot 1: I = 0.17, p = 0.01; Plot 2: I = 0.17, p = 0.01; Plot 3: 0.17, p<0.001; Plot 4: −0.04, p = 0.51; Plot 5: I = 0.37 , p<0.001 ). In Plot 1, 2 and 3 Moran's I values decreased towards zero when the patches reached a range of ca. 3 m; in plot 5 patch range was 6 m (Fig. 2b).

**Figure 3:**
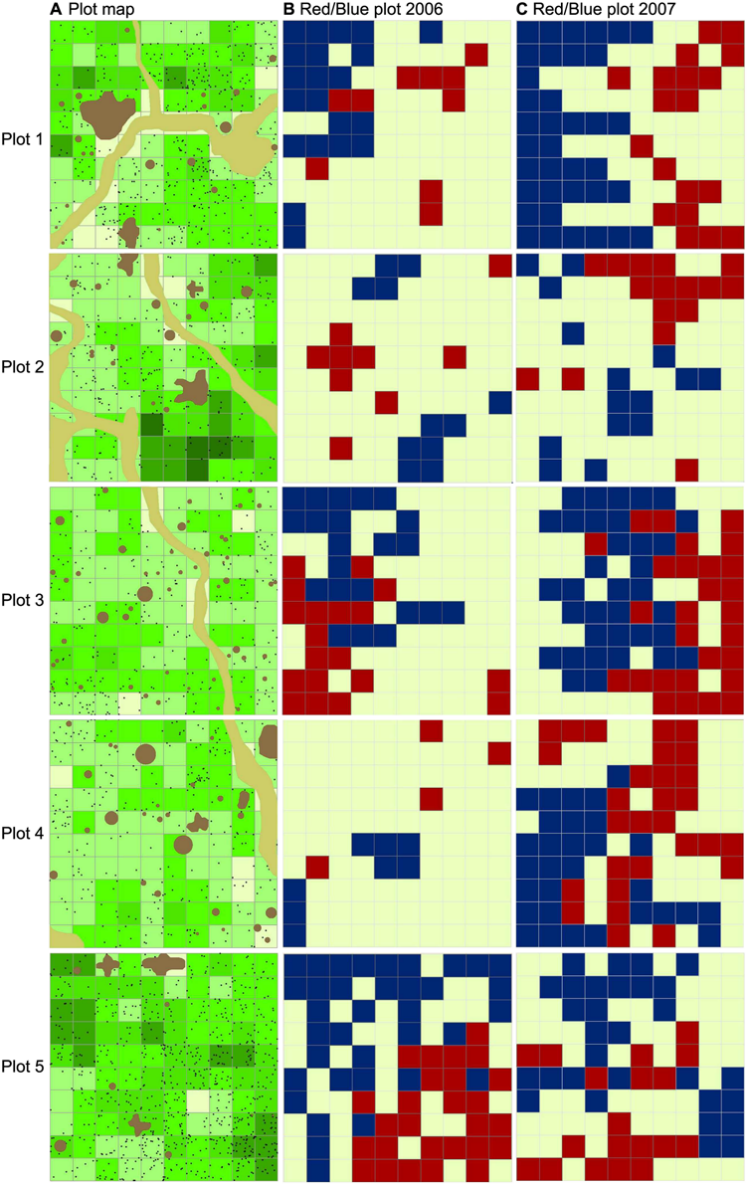
Red/Blue plots of the sampling plots. a. Schematic maps of the five 10×10 m sampling plots. Trees are shown in dark brown and paths in light brown. Black dots indicate cell densities of dead *O. unilateralis* infected ants (each dot is a single ant). Colour codes represent the vegetation cover of the cells, ranging from 0 (lightest green) to 5 (darkest green). b. Red/blue plot showing source cells in red and sink cells in blue in 2006. c. Red/blue plot showing source cells in red and sink cells in blue in 2007.

The test statistics from the SADIE analyses (Table 1) showed significantly clustered distributions in plot 1, plot 3 and plot 5 (I_a_>1.39, p<0.02), while distributions in plot 2 and plot 4 were not statistically different from random (I_a_<1.12, p>0.19). In each plot the means of local v_i_ and v_j_ values were nearly the same indicating that the plots are structured into sink (low *O. unilateralis* density) and source (high density) areas. However, the percentage of source area differed considerably between the plots. Plot 5 and plot 3 had the largest source areas (27% and 19%, respectively) followed by plot 1 and plot 2 (10% and 9%, respectively). Plot 4 had a source area of only 4%. A red/blue plot [22] depicting sources and sinks (Fig. 3b) identified a single large source area radiating from one corner in plot 5. Plot 3 contained one large and one small source area while the rest of the plots had several smaller sources scattered around the plot.

**Table 1:** Test statistics from the SADIE analysis of the five plots computed for 2006 and 2007.

2006								
Plot	I_a_	P_a_	mean v_j_	mean v_i_	P_j_	P_i_	% sink	% source
1	1.40	0.02	−1.38	1.33	0.02	0.04	21	10
2	1.12	0.2	−1.07	1.16	0.27	0.14	11	9
3	1.79	<0.01	−1.70	1.58	<0.01	<0.01	22	19
4	0.96	0.55	−0.97	0.94	0.52	0.60	8	4
5	2.46	<0.01	−2.18	2.15	<0.01	<0.01	36	27

2007								
Plot	I_a_	P_a_	mean v_j_	mean v_i_	P_j_	P_i_	% sink	% source
1	2.15	<0.01	−2.05	2.12	<0.01	<0.01	37	19
2	1.36	0.03	−1.40	1.31	0.02	0.04	5	16
3	2.00	<0.01	−1.95	1.71	<0.01	<0.01	35	28
4	1.94	<0.01	−1.90	1.93	<0.01	<0.01	30	29
5	1.72	<0.01	−1.67	1.51	<0.01	<0.01	30	19

I_a_ is an overall index of aggregation and P_a_ the associated probability value. P-values<0.025 indicates a statistical significant aggregated distribution (5%, two-tailed) and p-values>0.975 indicates a significant regular distribution (5%, two-tailed). V_i_ and V_j_ are indices for sources and sink areas, respectively, and P_i_/P_j_ values indicate the probability of obtaining a test value respectively exceeding or below the observed test value after 6000 randomizations. The last two columns reports the percentage of sink and source area in a plot.

### Spatial structure associations

At the largest scale, the occurrence of graveyards of dead *O. unilateralis* infected ants was not significantly correlated with temperature, humidity or light. At the smallest scale of 1 m^2^ the density of *O. unilateralis* in a cell did not seem to be strongly associated with either vegetation cover or the percentages of trees and path area in a cell. In the five plots correlations between cell counts of *O. unilateralis* and the measured variables were all weak and mostly non-significant. The only statistically significant correlations were found in plot 5 between *O. unilateralis* density and vegetation cover (Spearman, r_s_ = 0.18; p = 0.04) and in Plot 1 between *O. unilateralis* density and vegetation cover (Spearman, r_s_ = 0.25; p = 0.01) and percentage of path (Spearman, r_s_ = −0.22; p = 0.01).

At the intermediate scale, analysing the 4 m^2^ subplots, significant positive correlations were found between mean *O. unilateralis* density in the 25 subplots and vegetation cover (Spearman, r_s_ = 0.47; p<0.01), temperature (Spearman, r_s_ = 0.56; p<0.01) and absolute humidity (Spearman, r_s_ = 0.61; p = 0.01). A strong correlation was also found between temperature and absolute humidity (Spearman, r_s_ = 0.87; p<0.01).

### Temporal dynamics

The total recount of all cells in September 2007 revealed some dramatic changes in mean density and distribution in the plots (Fig. 3 b, c). In plots 3 and 4, the mean densities had more than doubled from 2.8 ants/m^2^ in 2006 to 6.6 ants/m^2^ and 5.8 ants/m^2^, respectively, in 2007 (Wilcoxon signed-rank test, Plot 3: Z = −6.81, p<0.01; Plot 4: Z = −6.22, p<0.01). Density frequency distributions were also much less skewed and had a considerable broader range than in 2006. After a year these plots had changed from low density, only slightly patchy plots to very structured, high density plots. In contrast plots 1, 2 and 5 had their mean densities reduced by 40–50% (Wilcoxon signed-rank test, Plot 1: Z = 4.64, p<0.01; Plot 2: Z = 4.66, p<0.01; Plot 5: Z = 6.91, p<0.01). Plot 1 from 5.0 to 3.1 ants/m^2^, Plot 2 from 3.3 to 1.7 ants/m^2^, and plot 5 from 8.2 to 4.3 ants/m^2^ and, compared to 2006, the frequency distributions had become more skewed in these plots. In all plots except plot 5 spatial correlograms showed increased levels of autocorrelation in 2007 (Fig. 2b) and the diameters of the clusters had increased from ca. 3 to 4–5 m, whereas cluster diameters in plot 5 had been reduced from 6 to 3–4 m. These trends were confirmed by the SADIE analyses, showing increased and statistically significant values of I_a_ in all plots except plot 5, as well as greatly increased sizes of clusters (Table 1, Fig. 3c). In plot 5, however, we found decreased, but still statistically significant, values of I_a_ and the area of clusters was also smaller than in 2006.

Comparing the distributional patterns from 2006 and 2007, SADIE's test for spatial association found a significant overlap of the spatial distributions of dead *O. unilateralis* infected ants in plot 1 (X = 0.49, p<0.01) and plot 5 (X = 0.27, p<0.01), indicating that patches had not shifted as much in location as in size in these plots. In the remaining plots new patches had emerged since no correlation could be found between the spatial structure in 2006 and 2007 (Fig. 3c).

**Figure 4:**
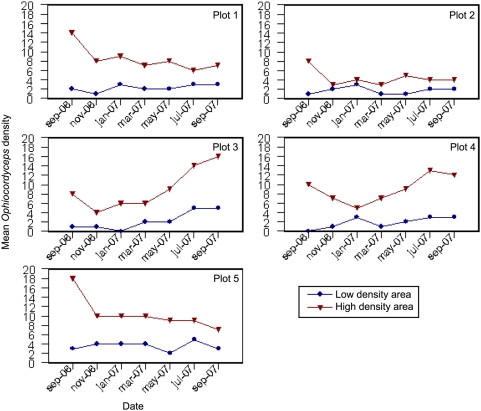
Temporal changes in *Ophiocordyceps* densities. Mean *Ophiocordyceps* densities for high density areas (red) and low density areas (blue) in each of the five plots from September 2006 until September 2007.

Overall we found that in just a single year the mean densities had changed radically within the study area. Some plots had become patchier and the existing patches had moved, whereas others showed the opposite trend, indicating that the distribution of dead *O. unilateralis* infected ants is spatially highly dynamic. The bi-monthly counts in high and low density patches within the plots further illustrated this. From the end of the rainy season in September until the end of the dry season in February, a steady decrease in mean *O. unilateralis* densities was observed. A more detailed look at the changes in *O. unilateralis* density between the bi-monthly counts (Fig. 4) showed that the heaviest losses (4–7 ants/m^2^) took place in high density patches reducing the mean densities with ca 45%, but that the changes in low density patches were relatively small. After the onset of the rainy season in May the mean densities increased again but only in low-density patches, whereas high-density patches remained more or less constant. Plot 3 and 4, which originally had the lowest mean densities, were exceptions. Here both high and low density patches seemed to have an increasing influx of dead ants from March to July. Despite these striking changes, the overall mean density of the survey area did not change noticeably (2006: 4.4 ants/m^2^; 2007: 4.3 ants/m^2^).

## Discussion

### Spatial pattern

As predicted, and using a range of approaches, we have shown that the distribution of dead *O. unilateralis* infected ants is both patchy and highly dynamic (Fig. 2, 3). Scattered in the forest, graveyards with dead *O. unilateralis* infected ants can be at least 20–30 meters in range. Spatial analyses on the plots indicate that graveyards are structured into distinct patches with *O. unilateralis* densities significantly above (sources) and below (sinks) average. The graveyards also showed considerable variation in mean densities of dead ants, but like the previously reported graveyard of *O. kniphofiodes* in the Amazon [16], [17] we recorded very high numbers of *O. unilateralis* with densities up to 26 ants/m^2^.

At the intermediate spatial scale (4 m^2^), the density of dead ants within graveyards did correlate significantly with humidity, temperature and vegetation. On a large scale the location of graveyards within the forest did not correlate with such factors while at the smallest scale (1 m^2^ ) some correlations between the density and vegetation or path cover was observed. This is correlative and not causational and experimentation would be useful is teasing the relative effects apart.

**Figure 5:**
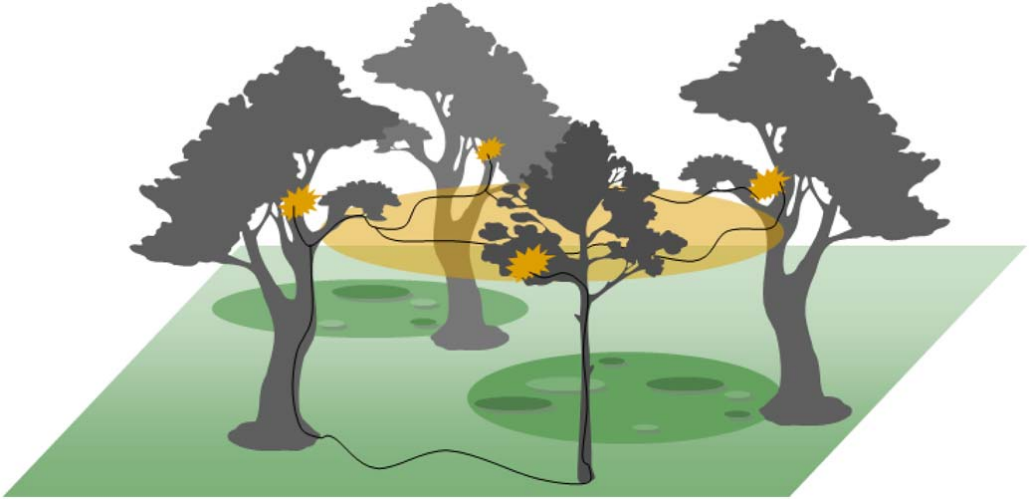
3-D habitat of the host ant *Camponotus leonardi*. A stylised representation of the 3-D habitat of the *C. leonardi* host ants, which includes both the high canopy and the understory. The normal foraging range of nests is shown by the orange shapes that represent focal nest sites and foraging areas, with the black lines representing trails across the canopy and onto the forest floor. The approximately 2-D habitat of the *Ophiocordyceps* killed ants in illustrated by the darker green ellipses representing graveyards. The aggregated distribution within graveyards has also been indicated.

An important factor to consider when examining the spatial patterns of dead ants is the location of live *C. leonardi* ants, their trails and their colonies. Despite extensive searching we only found two live *C. leonardi* ants in our plots, but more than two thousand dead ones. Trails of *C. leonardi* ants existed outside the plots but they were very rare; only one was found during September fieldwork both in 2006 and 2007 (ca. 5 m and 20 m from plots 4 & 3 respectively). Two additional trails were found in January 2007 (ca. 20 m from plot 3). We did determine that the principal host, *C. leonardi*, constructs its nests in the high canopy and has an extensive network of aerial trails (Figure 5 represents this). It is probable that aerial trails are the principal foraging routes and that the ants only occasionally descend to ground level since in all cases the ground level trails went back up into the canopy after no more than 3–5 m. Ants on these ground level trails were not observed foraging. We hypothesize that *C. leonardi* avoids the forest floor as a defence mechanism and only descends out of necessity when aerial trails cannot traverse canopy gaps. Further, resource profitability of a tree is potentially traded-off against the risk of losing workers due to infection when forest floor trails are the only way of access (cf Fig. 5). An interesting data point in support of this hypothesis comes from a disturbed forest nearby our site where no *O. unilateralis* exist: in that location *C. leonardi* trails were common at ground level (Anna M. Schmidt, personal communication). Experimentation and spatial ecology studies of the host ant are required to further test this hypothesis.

We have shown that graveyards exist and we also wanted to test if they were ephemeral as many examples of fungal disease in insects are [19] or rather more permanent features of the environment. Our data showed that graveyards are present in the same 100 m^2^ after 1 year and therefore not ephemeral. However, the distributional pattern of high density areas within graveyards is not static. The total recount within plots in September 2007 revealed a significant shift in the spatial distribution of dead ants with new low and high density patches becoming established and old ones disappearing. The density in a patch will be the result of local rates of disappearance and appearance of dead *O. unilateralis* infected ants. In general, for fungi that parasitize insects, appearance of new infected hosts is expected to be highest during periods with high humidity [23], [24], [25], [26]. The existence of high and low density patches within the graveyards suggests, however, that the emergence and disappearance rates of *O. unilateralis* are not uniform, but vary from patch to patch in both time and space.

*O. unilateralis* disappearance rates are likely to depend upon the rate at which ant cadavers decay. Cadaver disappearance is likely to be primarily affected by direct microclimatic conditions (affecting the desiccation level of the fungus) and indirect ones (the scavengers and decomposing microorganisms that colonize cadavers under these conditions). Our data (Fig 4) suggests that low density patches initially have low rates of both *O. unilateralis* emergence and disappearance. The subsequent increase where low density patches become high density ones is more likely a result of a higher rate of emergence than a lower rate of disappearance of dead ants relative to high density patches. While disappearance is likely to be due to many biotic and abiotic factors the emergence of dead ants in patches is related to the behaviour of infected ants prior to biting. The spectacular biting behaviour under leaves is evidence of parasite manipulation [16] but there is no evidence that choosing an optimal patch of forest in which to bite is adaptive manipulation. It may well be an emergent property of the system or a by-product of nest site location. Further work is needed to test these ideas as well as assessing the fitness benefits, if any, of patchy distributions for the fungus.

### Conclusion

Our study has shown that the extended phenotype of the parasitic *O. unilateralis* fungus has a strong effect on the spatial structure of dead ants; that the expression of these extended phenotypes interacts with environmental conditions; and that the parasite may even affect the foraging behaviour of the host. These findings re-enforce the need to explicitly consider the role that behaviourally manipulating parasites play in structuring the populations of their hosts and potentially of other species with correlated distributions. In the case of ants which are dominant and important members of tropical ecosystems [27], [28] the formation of graveyards may have ripple on effects that need to be explored.

## Materials and Methods

### Site Description

We situated our work in Thailand because the repeated and extensive collections of fungi by BIOTEC (www.biotec.or.th/Mycology) over the last 15 years have produced a herbarium with more than 12,000 accessions. This facilitates our work via a wealth of systematic, life history and natural history knowledge. Fieldwork took place in September 2006, ca. 20 km east of Trang in southern Thailand (7°32′49.50″N 99°47′14.73″E). Here a 24 ha Forest Dynamics plot (FDP) was established in 2000 on the North – North-eastern slopes of the hills in the Peninsula Khao Chong Botanic Garden as part of the Center for Tropical Forest Science (CTFS) pan-global Forest Dynamic Plot (FDP) initiative (www.ctfs.org). The site is exceptionally sandy, having both coarse and fine-grained sands and covered by a primary mixed evergreen forest, with an understorey dominated by saplings (<1 m). The climate is tropical with seasonal monsoons and mean monthly maximum temperatures range from 29.0°C to 33.4°C, peaking in March-April. Rainfall is heaviest from May to September and a dry season extends from November to February (climatic records taken at the site are available upon request).

Within the FDP a ca. 250×70 m survey area was delimited at an altitude of 120–140 m. Here several areas (graveyards) with dead *O. unilateralis*-infected ants were found in which four 10×10 m sampling plots were established. Plot 1 and plot 2 were located in an area dominated by several large buttress trees, with a dense canopy and well traversed with foot paths used by forest workers. Plot 3 and plot 4 were situated ca 50 meters downhill between a stream and a small path. Further downhill, a few hundred meters north of the FDP, a fifth 100 m^2^ sampling plot was marked out (plot 5) in an area with a more open canopy. Within the 250×70 m survey area, but away from the plots, two parallel 200 m transects, 40 m apart, were also laid out, with 2×2 m plots at 20 m intervals.

We systematically turned over and checked the underside of all leaves in both sampling plots and transect plots, up to a height of 2 m (preliminary and subsequent sampling has never recorded dead ants above 2 m, details available upon request). We only checked under leaves because the extensive collections by BIOTEC have established that *O. unilateralis* killed ants are only found in this habitat. The existence of an already established FDP made accurate georeferencing (i.e. spatial mapping) of dead ants possible since GPS does not function under forest canopies. It further allowed the use of geostatistical tools for the analyses of the distribution of dead *O. unilateralis* infected ants. We used a series of baiting and trapping methods to capture live ants. Since live ants of the principal host (i.e. *Camponotus leonardi*, see results) were rarely found we employed extensive searching for lone individuals and trails. We also climbed into the canopy searching for live ants of the principal host species.

### Data Collection

The transect plots and the five 100 m^2^ sampling plots were divided into 1 m grids creating 1 m^2^ cells. All cells were georeferenced and the number of dead *O. unilateralis* infected ants counted. Vegetation cover was also estimated per cell and scaled from 0 (bare ground) to 5 (very dense vegetation). In all plots humidity and temperature were measured ca. 30 cm above ground (this corresponds to the average height at which ants bite the leaf, S.B. Andersen *et al*, unpublished data). Each recording lasted 15 minutes with one reading every minute. In the transect plots, one recording event was taken in the middle, while in the sampling plots 5 records were taken - one in the middle of the 100 m^2^ plot and one in each corner. All recordings were conducted between 1000–1600 hrs. In the transects we also measured light intensity ca. 30 cm above ground. In the five sampling plots trees and paths were mapped and the percentages of ground covered by paths or trees were estimated for each cell.

Since fungal disease outbreaks in plants and insects are often ephemeral phenomenon we performed counts over time. We chose four high density cells and four low density cells from each of the five sampling plots, i.e. 40 cells, following the initial survey in September 2006. These cells were recounted in November 2006, and then January, March, May and July 2007. In September 2007 a total recount was performed on all 500 1 m^2^ cells in the five plots. Thus during 12 months we examined every leaf and counted the number of dead *O. unilateralis* infected ants in 1,360 m^2^ of primary rainforest.

### Spatio-temporal analyses

Two different methods were used for georeferenced count data in order to evaluate the spatial structure. First, we constructed Moran I correlograms for both transects and sampling plots to assess trends in the spatial autocorrelation of dead *O. unilateralis* infected ants. For these analyses we used the statistical software SAM v2.0 [29] freely downloadable at www.ecoevol.ufg.br/sam.

Second, we further analysed the pattern and degree of patchiness in the spatial structure with the use of SADIE (Spatial Analysis by Distance IndicEs), also freely downloadable at www.rothamsted.ac.uk/pie/sadie/SADIE_home_page_1.htm [30]. The transect data were neither suited nor relevant for this kind of analysis, hence only data from the plots were analysed.

SADIE computes an index of aggregation I_a_ on the basis of the minimum distance that individuals in a sampling grid would have to move between cells for all cells to contain the same number of individuals (distance to regularity). A spatially random sample will give I_a_ = 1, while I_a_<1 indicates a regular distribution and I_a_>1 an aggregated distribution. Random permutations of the cell counts provide a method for statistical testing [30]. In addition local indices of “patchiness” were computed for each cell. The local indices v_i_ and v_j_ denote the degree to which a cell must receive or deliver individuals in order to obtain regularity. Values of v_i_ higher than ∼1.5 indicate that the cell belongs to a source (cluster) while v_j_ values lower than ∼−1.5 indicate association to a sink (gap). Values around zero represent randomness [22].

SADIE was also used to evaluate the temporal changes in spatial distribution between 2006 and 2007 [31] by calculating the X-statistic (i.e. the correlation coefficient between the clustering indices of the two sets). Randomisation tests allowed for assessment of the significance of this statistic (X) with p-values<0.025 indicating a significant association between datasets (5%, two-tailed) and p-values>0.975 indicating a significant disassociation between datasets (5%, two-tailed).

### Spatial structure associations

In order to detect associations between the densities of *O. unilateralis* infected ants and environmental factors operating at small, medium and large scales, analyses were carried out on three subsets of data. For the large scale the data from the 200 m transects were used to correlate the mean *O. unilateralis* densities in the 4 m^2^ transect plots with environmental factors such as light, humidity, vegetation cover and temperature across the landscape. For analysis on an intermediate scale, 4 m^2^ subplots were established around the humidity/temperature measuring points within the 10×10 m sampling plots giving 5 such subplots in each of the five 10×10 m plots. Mean values of *O. unilateralis* density, percentage path coverage, percentage tree and vegetation cover and measurements of temperature and humidity were used to assess correlations within *O. unilateralis* graveyards. On the smallest scale, cell counts of *O. unilateralis* density and measurements of overall vegetation cover and percentage tree and paths cover were used to analyse correlations across cells in the five plots. Correlation analyses on transects and plots were controlled for spatial autocorrelation using SAM v2.0. To control for family-wise Type I errors when doing multiple correlational analyses a sequential Bonferroni procedure was used to adjust significance levels [32].
